# Racial/Ethnic Disparities in Chronic Diseases of Youths and Access to Health Care in the United States

**DOI:** 10.1155/2013/787616

**Published:** 2013-09-23

**Authors:** James H. Price, Jagdish Khubchandani, Molly McKinney, Robert Braun

**Affiliations:** ^1^Health Education and Public Health, University of Toledo, Toledo, OH 43606, USA; ^2^Community Health, Ball State University, Muncie, IN 47306, USA; ^3^Public Health, Eastern Kentucky University, Richmond, KY 40475, USA; ^4^Health Sciences, Otterbein University, Westerville, OH 43081, USA

## Abstract

Racial/ethnic minorities are 1.5 to 2.0 times more likely than whites to have most of the major chronic diseases. Chronic diseases are also more common in the poor than the nonpoor and this association is frequently mediated by race/ethnicity. Specifically, children are disproportionately affected by racial/ethnic health disparities. Between 1960 and 2005 the percentage of children with a chronic disease in the United States almost quadrupled with racial/ethnic minority youth having higher likelihood for these diseases. The most common major chronic diseases of youth in the United States are asthma, diabetes mellitus, obesity, hypertension, dental disease, attention-deficit/hyperactivity disorder, mental illness, cancers, sickle-cell anemia, cystic fibrosis, and a variety of genetic and other birth defects. This review will focus on the psychosocial rather than biological factors that play important roles in the etiology and subsequent solutions to these health disparities because they should be avoidable and they are inherently unjust. Finally, this review examines access to health services by focusing on health insurance and dental insurance coverage and access to school health services.

## 1. Background

 More than 133 million Americans (45% population) have one or more chronic diseases. Racial/ethnic minorities are 1.5 to 2.0 times more likely than whites to have most of the major chronic diseases [1]. Chronic diseases account for 7 in 10 American deaths and account for 78 cents of every health care dollar spent [1, 2]. Unfortunately, the problems of chronic diseases seem to be getting worse. For example, between 1960 and 2005 the percentage of American children with a chronic disease almost quadrupled (from 1.8% to 7.0%) with racial/ethnic minority youth being affected disproportionately [1]. Increased rates of childhood chronic diseases imply that higher rates of these illnesses will occur during adulthood.

 The prevalence of chronic diseases in childhood is highly variable based on the conceptual definitions and operationalization of the concept used in the various studies [3]. However, the general rule of thumb is that chronic diseases in childhood are more common in boys, older youths, those from low-income families, and racial/ethnic minorities. For adolescents, the best estimate would be that 15% to 35% of these youths have one or more chronic diseases with racial minorities being affected disproportionately [3, 4]. The most common major chronic diseases of youths in the US are asthma, diabetes mellitus, obesity, hypertension, dental disease, attention-deficit/hyperactivity disorder (ADHD), mental illness, cancers, sickle-cell anemia, cystic fibrosis, and a variety of genetic and other birth defects. This range of childhood chronic diseases and the health disparities that exist for most of these diseases indicate that both biological and psychosocial factors play important roles in the etiology and subsequent solutions to these health disparities. This review will focus more on the psychosocial disparities in health of US youth because they should be avoidable and they are inherently unjust [5].

## 2. Asthma

 Asthma is the most common chronic disease of childhood [6]. Asthma is a chronic inflammatory disorder of the airways, characterized by shortness of breath, coughing, wheezing, chest tightness caused by airflow obstruction due to allergic sensitization, and airway hyperactivity. It is not known why some youths get the disease and others do not. The majority of children with asthma will have recurrent attacks, usually reversible with appropriate treatment or abates spontaneously. Some children have mild attacks and others have severe life threatening attacks. Asthma attacks are usually triggered by a wide variety of things, including allergens (e.g., cockroaches, dust mites, pollen, etc.), exercise, changes in weather, air pollutants (e.g., tobacco smoke), stress, infections, diet, and obesity [6–8].

 In 2007, 13.1% of children (9.6 million) had, at some time during their lives, a diagnosis of asthma and 9.1% (6.7 million) currently had asthma [9]. Asthma prevalence is higher in the Northeast and Midwest areas of the US [10]. The prevalence of asthma is higher in older children (ages 11 to 17 years), those who are poor, and in boys. The highest rate of asthma is found in Puerto Rican Americans (13.1%), followed by African Americans (9.5%), whites (7.2%), and Mexican Americans (3.6%) [11]. Considerable disparities exist for asthma care for African Americans compared to white youths. African American youths have emergency department (ED) visits four times that of whites, three times the hospitalization rate, and 7.6 times the death rate [9]. However, African American youths use nonemergency physician office visits for asthma at a rate 20% lower than for white youths [9]. Recurrent ED visits and hospitalizations significantly increase the risk for subsequent fatal asthma attacks. In 2008, 10.5 million school days were missed due to asthma [10].

Most researchers in asthma acknowledge that asthma may be a complex of allergic sensitization diseases that are genotypically and phenotypically different across individuals and groups [11]. The complex nature of asthma means that a plethora of things have been found to be associated with the incidence of asthma. Low socioeconomic status youths have a 50% increased risk of developing significant wheezing problems [12]. Low birth weight is more common in African Americans and has been found to be a risk factor for asthma. Studies have found that 30%–68% of the racial differences in asthma could be explained by low birth weight [13, 14]. Children from poor neighborhoods often spend less time outdoors because of an absence of parks, playgrounds, and recreational programs and because of potential safety issues [15]. Thus, these youths are likely to spend more time indoors, exposed to indoor allergens, and being more sedentary these youths are likely to gain excessive weight.

 Psychosocial factors are also associated with an increased incidence of asthma. Economically disadvantaged neighborhoods are characterized by concentrated poverty, widespread unemployment, substandard housing, inability to pay utilities, eviction notices, high crime and violence rates, and lack of social supports and control over their lives [16]. One study has shown increased asthma symptomology with increased exposure to violence (adjusted for SES and negative life events). Stress is higher in residents of economically disadvantaged neighborhoods and stress exacerbates asthma morbidity. Mistrust of the health care arena by many African Americans also impedes quality of asthma care. A study of Hispanic and African American parents of children with asthma found that 89% had treated their children with some form of complementary or alternative medicine, and most did not inform the child's physician [17]. In addition, 59% believed the alternative therapy was as effective as the physician prescribed medications. This may be related to a qualitative study of African American parents that noted parent concerns regarding medication side effects and the possibility that their children might become addicted to asthma medications [17]. 

 The final critical issue associated with racial/ethnic disparities in asthma morbidity and mortality is access to and quality of health care. Fragmented and episodic care from multiple health care providers is characteristic of minority urban children [18]. Another clinical problem is that racial/ethnic minority children are less likely to be given prescriptions for preventive asthma medications [19]. Additionally, this poorer quality of care is more likely to occur in community health centers and hospital clinics, two regular sources of care for many urban minority youths [20]. Finally, the growing economic burden to states caused by the Medicaid population has caused many states to introduce copayments, which are negatively affecting the poor. One study found that by doubling existing copayments it resulted in a 21% decrease in the number of prescriptions filled for asthma [21].

## 3. Diabetes Mellitus

 Diabetes mellitus is a group of metabolic diseases associated with regulation of blood glucose. The etiology of diabetes varies by type of the disease but includes genetic, environmental, and behavioral factors. There is also a condition known as prediabetes, where elevated blood glucose levels exist and the person with this condition is highly likely to develop diabetes. About 24 million Americans have diabetes and 57 million have prediabetes [22]. The national economic burden of these conditions reached $218 billion in 2007, representing 7% of the $2.24 trillion in national health care expenditures [23]. Between 2009 and 2034 the number of Americans with diabetes is expected to reach 44 million and the costs will likely rise to $336 billion [24].

 There are two main forms of diabetes mellitus; type 1 diabetics do not produce insulin and must receive exogenous insulin through injections or an insulin pump. The majority of school-aged youths have historically had type I diabetes. The second form of diabetes, type 2, is the most common form of the disease and has been most often diagnosed in obese adults [25].

Youths and adults with type 2 diabetes may be able to control their disease through diet and exercise or it may require the addition of oral medications or insulin injections.

 The incidence and type of diabetes vary by age, gender, and race/ethnicity. The incidence of type 1 diabetes in white youths is 24.5/100,000 for males and 22.7/100,000 for females. Type 2 diabetes is rare in white youths before 10 years of age. However, the incidence of type 2 diabetes is 3.7/100,000, with similar rates for both males and females [26].

 A major change is occurring regarding the types of diabetes being diagnosed in adolescents. In 1990, about 3% of all new cases of diabetes in youths were type 2, but by 2005 45% of all new cases of diabetes in youth were type 2 [27]. The most logical explanation for the increasing number of type 2 diabetes is the growing level of obesity in youths and the decline in exercise behaviors. The aforementioned study of white youths, depending on age, found that 9–12% of type I diabetics were obese but in type 2 diabetes 75–80% were obese [26]. A significant portion of these youths also had other cardiovascular and behavioral (e.g., smoking) risk factors.

 In contrast to white youths the incidence of type 1 diabetes in African American youths was 15.7/100,000 and the incidence of type 2 diabetes was 19.0/100,000 [28]. Type 1 diabetic African American youths had a prevalence of about 20% obesity, whereas about 80% of type 2 diabetics were obese. Poverty was also associated with youths who had both type 1 diabetes (33%) and type 2 diabetes (60%) [28].

 Among Hispanic American youth, type 1 diabetes is more common than type 2 diabetes. For younger (ages 10–14 years) Hispanics, 62% had type 1 diabetes and 38% had type 2, while for those of 15–19 years of age, 42% had type 1 and 58% had type 2 [29]. The incidence of type 1 diabetes was 14.1/100,000 for females and 15.6/100,000 for males. For type 2 diabetes, the rates were 6.9/100,000 and 4.8/100,000, respectively. With regard to overweight and obesity, 15–20% of type 1 Hispanic youths fell in this weight range and 70–75% of type 2 Hispanic youths were in this weight range [29]. About 35% of type 2 Hispanic youths lived in poverty, as did 20% of type 1 Hispanic youth. Over 50% of diabetic Hispanic youths had a family history of diabetes.

## 4. Obesity and Overweight

 Childhood obesity is a chronic public health problem that is growing at an increasingly rapid rate. Studies have found that approximately 16% of youth are overweight and 32% are obese; this represents a threefold increase, in comparison to rates in the 1960s [30, 31]. Obesity and overweight prevalence vary by race/ethnicity; 13% of white youths, 24% of African American youths, and 23% of Hispanic youths are obese. Whereas 27% of white youths, 41% of African American youths, and 41% of Hispanic youths are overweight [32]. This increase in body weight has numerous implications for obese youth that are likely to have their obesity track into adulthood. Obesity at virtually any level has been associated with multisystem stress not seen in those of regular body weight [33]. Rates of overweight/obesity are 3-4 times higher for those children coming from households with low income and low parental education [34].

 Obesity is a multifaceted problem; there are many factors that play a role in childhood obesity. Low socioeconomic status, physical inactivity, skipping breakfast, high consumption of sweetened beverages, irregular sleep patterns, having low personal weight concerns, peer influences, and chronic health conditions all play roles in the rate at which children will become overweight or obese [35–37]. Racial and ethnic minorities are at a disadvantage for becoming overweight as children and remaining so into adulthood because most of the aforementioned factors impact them more than white youths.

 The lack of physical activity also puts children at a higher risk for obesity. School-aged children are at a disadvantage for having adequate physical activity incorporated into their day. Often schools have limited time allowances for physical education and inadequate funding for physical exercise equipment [38]. In addition, fewer elementary schools have recess or physical education. Due to these factors, it has been found that physical activity for adolescent girls decreases at a rate of 4% per year from the sixth to the eighth grades [39]. 

 Nutrition is another factor that influences overweight and obesity. Children coming from food-insecure homes, meaning that food was not always available to them due to economic limitations, were more likely to have poor nutritional habits by not eating breakfast and consuming large amounts of fast foods and few family prepared meals [40]. In addition, a study of multiple communities found that wealthy neighborhoods had three times the number of grocery stores as low socioeconomic neighborhoods, while white neighborhoods had four times the number as African American neighborhoods [41]. African Americans living in a census track with one or more supermarkets are more likely to consume fruits and vegetables than African Americans living in neighborhoods without supermarkets. Furthermore, for every additional neighborhood supermarket, produce consumption increased by 32% [41]. 

Families that has low socioeconomic status do not have access to fruits and vegetables at the same rate as higher income families [38]. It has been found that African American students from low socioeconomic status families often consume significantly more empty calories in the form of sugars and fried foods and they feel there is nothing they can do to change their dietary situation [39]. 

 All of the aforementioned factors work together to make US children some of the most overweight/obese in the world. Prevention of this ever growing problem must be comprehensive in addressing the causative factors for excess weight gain. Making education culturally relevant to the target population is of the utmost importance [40]. Reduction of socioeconomic disparities, racial segregation, and stress facing low-income families can also greatly reduce the rate of racial/ethnic childhood obesity disparities [42, 43]. Increasing available healthful food choices in schools (e.g., school breakfast and lunch programs) and the general environment will aid in increased health benefits and lowering of childhood overweight. 

 If the epidemic of childhood obesity is not curbed, society will reap the long-term consequences. Overweight children have a greater propensity to become obese adults. As adults, they will have higher rates of coronary heart disease, type 2 diabetes, and muscle-skeletal issues and be subject to more discrimination [44–47]. The costs of these diseases in adulthood will exponentially increase the amount spent on health care and society will have less resources for other expenditures (e.g., schools) [48]. Obesity related diseases result in approximately 95 million years of life lost [49]. These years lost are due to chronic conditions caused by overweight and obesity which are preventable. 

## 5. Hypertension

 Hypertension can be present at any age, including infancy. Approximately 5 of every 100 children have higher than normal blood pressure (prehypertension) but less than 1 in 100 has significant hypertension. High blood pressure is when a child has the same or higher than the ninety-fifth percentile for children who are of the same age, sex, and height [50]. What is considered normal pressure varies by age.

 Conditions that increase the risk of hypertension include history of prematurity, very low birth weight, congenital heart disease, kidney disease, obesity, lack of adequate exercise, sleep apnea (associated with overweight), smoking, illegal recreational drugs (e.g., steroids, cocaine, etc.), and a family history of hypertension. Most child hypertension is secondary to one or more of the aforementioned conditions [51].

 Recent research indicates that for normal weight boys, African Americans were significantly more likely than whites (OR = 1.14) and significantly more likely than Hispanic boys (OR = 1.18) to have high blood pressure [52]. For overweight boys, Hispanics were more likely than whites (OR = 1.23) but overweight African Americans were not more likely than whites to have high blood pressure. For girls of normal weight, African American girls were more likely than white girls to have prehypertension (OR = 1.32), but no difference was found for Hispanic girls. High blood pressure was more common in African American girls than white girls (OR = 1.17), but the difference became nonsignificant after adjusting for body mass index [53]. A recent study of hypertension in children found that African American children with hypertension have an increased prevalence of obesity, left ventricular hypertrophy, and more pronounced blood pressure elevations than white children [54]. There is a tendency for youths with hypertension to have their disease track into adulthood. 

## 6. Mental Illness

The prevalence of mental illness in US children is poorly documented [55]. In general, it is reported that depression, anxiety disorders, attention-deficit/hyperactivity disorder (ADHD), and substance abuse disorders are the most common mental health problems of youth in the US [55, 56]. However, according to various estimates, at any given time, 10%–21% of children in the United States have a diagnosable mental illness resulting in functional impairment [57–59]. Additional prevalence estimates of mental illness range from 17.6 to 22% in one study [60]; to 16–18% in another [61]. A recent web update by the American Academy of Child and Adolescent Psychiatry reported that conduct disorder affects 1 to 4% of 9- to 17-year-old people, 10% of children and adolescents are affected by serious emotional disturbances, and 6% of the school-age population has oppositional defiant disorder [62]. Additionally, a Surgeon General report documented the prevalence of mental illness in US children as 13% for anxiety disorder, 6% for mood disorders, 10% for disruptive disorders, and 21% for any mental health disorder [63]. Furthermore, it was reported that child mental disorders persist into adulthood; 74% of 21-year-old people with mental disorders had prior problems [63]. If left untreated these mental health disorders often get worse, often becoming debilitating. In the United States, between the ages of 1 and 19, it is reported that the groups of conditions that have lower quality of life and reduce life chances the most are emotional and behavioral problems, in part, due to the associated social and functional impairments [63]. One of the modern trends is that youth in the US are also battling mental disorders at increasingly earlier ages with the onset of half of all psychiatric disorders occurring by age 14 [64]. 

 Race specific prevalence estimates for mental illnesses in youth are scant in the literature. A Surgeon General's report concluded that rates of mental disorders are similar between whites and minority populations in the general community [63–66]. However, the broadest and the most comprehensive national indicator of any mental health outcome for youth is estimated by the Youth Risk Behavior Surveillance System (YRBSS). The only mental health outcome assessed nationwide and periodically is the presence of “depressive symptoms.” The YRBSS has a specific question for youth “Have you ever felt so sad or hopeless almost every day, for two weeks in a row, that you couldn't do some of your usual activities?” According to the YRBSS 2009, 23.7% of white youths reported having felt sad or hopeless almost every day for 2 weeks in a row or longer compared to 27.7% of black youth and 31.6% Hispanics. Proxy measures like suicide, substance abuse, and other violent behaviors are often discussed in the context of mental health. The 2009 YRBSS found that Hispanic youths had the highest rate of suicide attempts of any racial/ethnic group, a proxy measure for behavioral and emotional problems [67]. 

Minority status is a predictor of youth psychopathology. There are several causative mechanisms for race being a predictor of youth psychopathology and the associated disparities in youth mental health status based on race/ethnicity [68–71]. Poverty leads the discussion in research as it relates to poorer mental health status and outcomes for minority youth. Minority youth disproportionately live in a chronic state of poverty or severe poverty, which has cumulative effects on mental health. Poverty has direct effects on adolescent mental health, quality of life, and social judgment. Exposure to poverty increases the risk for mental disorders such as depression and behavioral risks such as substance abuse and criminal activity [71–73]. Another closely tied factor to mental health is household structure and parenting. Brody and colleagues demonstrated that the likelihood of developing conduct disorders among African American youth was associated with parenting and household factors [74]. Lack of health insurance and other access to care issues for mental health services largely affects minority youth. Even though a variety of mental health service usage factors in racial/ethnic minorities are related to poverty, minority individuals experience greater barriers to access even when age, education, and income factors are controlled, suggesting that additional factors have to be considered [75].

 Prevalence of mental health service use in youths varies from 6% to 7.5%, indicating that about one-third of all youths who need mental health services actually receive them [66]. Additionally, children with public insurance are more likely (9–13%) than privately insured (5–7%) or uninsured (4-5%) youths to obtain mental health services. In the National Health Interview Survey, 80% of African American youths, 82% of Hispanic, and 72% of white youths with mental health issues had unmet mental health care needs [75]. Increasing the use of mental health services and improving the quality of care delivered to them will be helpful in bridging the gap as it relates to actually improving racial/ethnic youth mental health by solving the social determinants (e.g., prejudice, stress, socioeconomic status, quality of education, etc.) which increase the vulnerability of these youths.

## 7. Attention-Deficit/Hyperactivity Disorder

 Attention-deficit/hyperactivity disorder (ADHD) is primarily a genetic disorder (76%) characterized by inattentiveness, impulsivity, hyperactivity, and functional impairments (e.g., impaired academic success) [76]. ADHD is one of the most common childhood psychiatric conditions, affecting approximately 7% of youths [77]. When parents and teachers were given specific criteria representing characteristics of ADHD, African American children were more likely to be rated higher on these criteria. However, white children are 1.5 times more likely to be treated for ADHD [78]. It has been reported that Hispanics are about one-half as likely (3.3%) as whites to have ADHD but are one-third as likely to be treated for ADHD [79, 80]. Disparities in treatment by race/ethnicity remain even after socioeconomic factors are controlled for in the analyses [81].

 Youths with a diagnosis of ADHD frequently have comorbid psychiatric disorders, including oppositional defiant disorder (ODD) (54%–84%), anxiety disorders (up to 33%), depressive disorder (up to 33%), and increased rates of smoking (300%) [77, 82]. For those youth who have their ADHD continuing into adulthood (60%) their ODD will likely evolve into antisocial personality disorder and their psychopathology will increase as young adults [77].

 Treatment for ADHD will reduce functional impairments of youths with this disorder. The primary goals of therapy for ADHD, using stimulants and behavioral treatments, are to improve academic performance, decrease disruptive behaviors, improve impulse control, and improve family and peer relationships. Racial/ethnic minorities with ADHD have shown the best treatment outcomes when both forms of care are provided [77].

 Research indicates that there are disparities in the rates of treatment for ADHD between affluent versus economically disadvantaged youths and between white and racial/ethnic minorities [83]. There are a multitude of reasons for these disparities, including affluent families having a stronger emphasis on academic success, African American and Hispanic parents knowing less about ADHD than white parents, African American parents worrying that drug therapy for ADHD is simply schools trying to make their children conform to their standards, and issues of access to health care [83]. 

 The disparities in treatment of ADHD raise a social justice issue, because affluent white youths are receiving cognition-enhancing drugs (e.g., stimulants) more than poor and racial/ethnic minorities [83]. ADHD is recognized as a “legal disability”; thus the affluent and white youths who have certain rights and academic accommodations are likely to increase their success than the poor and racial/ethnic minorities. ADHD recognized as a disability under the Americans with Disability Act affords such things for those students as a personal note taker, special times for tests, and extra time to take tests. In addition, at the K-12 grades, since ADHD is recognized as a learning disability, these students are entitled to special education services. This is likely to mean these students will be in smaller classes and have individual tutoring and extra time to complete academic assignments [83]. All of these accommodations mean that affluent and white students with ADHD are more likely to be successful than poor and racial/ethnic minority students with ADHD.

## 8. Insurance Status and Access to Health Services

 This section regarding racial/ethnic disparities addresses access to health services by focusing on health insurance, dental insurance, and school health services. The concept of access to health services needs to be conceptually expanded to include not just financial access (health insurance) but also appointment accessibility, how long it takes to get an appointment, and whether the physician has weekend or evening hours [84]. Many blue collar jobs are such that if you have to take off from work to take your child to the doctor you do not get paid for missing work. It is not uncommon for two-thirds of clinicians to have no weekend or evening hours [84]. Geographic access is another potential limitation to receiving care especially for those in the inner city. Many racial/ethnic minorities live in enclaves of lower socioeconomic status, areas often times perceived as less safe and less desirable for locating a medical or dental practice. Cognitive accessibility is a form of health literacy of knowing when to access health care and what type of care you should pursue. Overall accessibility relates to whether providers accept new patients in general and, more specifically, whether they accept Medicaid, CHIP, or a particular form of private insurance. For example, a recent study found that about 87% of physicians in a particular area were accepting new patients, but only 68% were accepting new Medicaid patients [84].

## 9. Health Insurance Coverage

 American youths (78.7 million) usually receive their health insurance, if they have health insurance, from primarily one of three sources: employers (also called private) insurance (54%) or one of two public insurance programs (Medicaid (25%) or Child Health Insurance Program (CHIP) (6%)) [85]. A variety of other sources (e.g., military healthcare, Indian Health Services, and individually purchased plans) insure a smaller segment (5%) of the child population. That leaves 10% of the child population (almost 8 million) without health insurance coverage [85]. Children have been rapidly losing coverage from employer insurance, where in 2000 66% of children were covered by this type of provider [86]. 

 The racial/ethnic composition of the uninsured children population is 39% white, 38% Hispanic, 16% African American, and 7% other [85]. It is likely that the lower participation rate in health insurance programs for Hispanic children is due to many Hispanic children being more likely to have noncitizen parents, which is known to be associated with lower Medicaid participation [87]. Of all the uninsured children, 64% are eligible for enrollment in Medicaid (41%) or CHIP (23%) [87]. This means that 88% of all low-income (less than 200% of the federal poverty level) uninsured children could be enrolled in one of these two programs. In fact, 40% of these eligible children were enrolled in one of the programs during the previous year, indicating a problem with retention of eligible children [88]. If we were to have full participation in Medicaid and CHIP by the children who are eligible we would reduce the number of uninsured African American children by 77%, Hispanic children by 65%, and white children by 60% [87].

 Medicaid and CHIP health insurance coverage for previously uninsured children not only increases access to clinical care but it increases access to prescription medications and dental care. Covered children have fewer unmet health care needs, are more likely to receive timely diagnoses of serious health conditions, and tend to have fewer avoidable hospitalizations [89].

 Few studies have specifically examined racial/ethnic differences in prescription drug use among children. A study of the 1996 Medical Expenditure Panel Survey (MEPS) found that African American children used one-third (33%) less prescriptions than white children, and uninsured children used almost 40% fewer prescriptions than privately insured children [90]. Children insured by Medicaid used just as many prescription medications as privately insured children, again, further evidence of the role played by public health insurance in helping to reduce health care disparities. Lack of health insurance results in inadequate access to health care, regardless of race/ethnicity [89]. However, while health insurance coverage reduces racial/ethnic disparities in access to care, disparities in accessing care continues to exist among both insured and uninsured children. Thus, health insurance is necessary but not sufficient to eliminate disparities in access to care which are more likely to affect children of color. These additional barriers may include transportation problems, problems with geographic access to health care providers, inadequate consumer health literacy of parents/guardians, and the need for more translation services [90].

 There are a number of options that could be pursued to provide more comprehensive health insurance coverage for children. It is essential that states reduce the cumbersome paperwork for enrollment and renewal of eligibility and reduce confusion regarding eligibility requirements. A study of low-income parents found that 91% with uninsured children who knew of Medicaid/CHIP programs claimed they would enroll their child if they knew he or she was eligible. However, 45% did not think their children were eligible, 55% did not know how to enroll their children in the public insurance programs, and 50% perceived the enrollment process difficult [87]. Another solution, which would require a significant change in federal legislators mind set, is to create a universal insurance program to cover all youths to age 21. In other words, a Medicare-like federal program under which all children would be automatically enrolled, regardless of family income. This would need to be a federal program because many states could not afford this increased financial burden. In fact, a disproportionate share of the currently uninsured Medicaid and CHIP children resides in the South, in part an indication of the state governments economic problems in those areas [91]. An additional change that would be beneficial would be to improve reimbursement rates to providers to ensure that the public health insurance program would provide for real access to health care. In addition, tax incentives could be provided to health care professionals to locate their clinic offices in underserved urban environments.

 Another option for increasing enrollment in Medicaid and CHIP is for schools to work with their state Medicaid and CHIP offices to help enroll eligible students. A national study of state CHIP directors found that about three-fourths (78%) were currently working with some schools [92]. The directors perceived there would be numerous benefits from working with schools for students and schools, including reducing student absenteeism, improving student attention and concentration, and reducing the number of students being held back in schools because of health problems. However, such working relationships will work only if school health service personnel and school administrators are supportive of such activity. A recent national study of school health nurses found that about one-third (34%) reported their schools expected them to help parents enroll their children in public health insurance programs [93]. A follow-up study to the school health nurses study on school superintendents found that the majority (52%) believed that schools should play a role in helping students enroll in public health insurance [94]. However, the majority of superintendents also cited two main barriers to being more involved in helping students obtain health insurance: not enough staff (62%) and not having financial resources (55%). Both of these barriers could be reduced by schools working with their state Medicaid/CHIP offices.

## 10. School-Based Health Care 

Schools have a vested role in having children who are healthy, not absent from school, and academically successful. These qualities of adolescents are in part, associated with having adequate access to health care. Chronically ill children are likely to miss more school days and to be less academically successful [95–97]. Schools currently provide a variety of services to help students more easily access health care, including mental health counseling, school-based health clinics (SBHCs), and school health services. Schools are commonly referred to as the *de facto* providers of mental health services for adolescents and were examined in the section on mental health issues [98].

 School-based health clinics (about 1500) started in the early 1970s and most (73%) have spread to urban schools, offering comprehensive health care services to students who might not otherwise have access to primary health care [99]. SBHCs typically provide access to preventive care, routine assessments and screenings, early interventions for both medical and behavioral problems, and assistance managing chronic illnesses. Two-thirds of the students served by SBHCs are racial/ethnic minorities. These centers are usually operated by hospitals or community health clinics. Advocates of SBHCs claim that these clinics help reduce geographic accessibility problems and reduce financial access impediments to needed health care for economically disadvantaged urban youths. Actual research that shows a significant effect of SBHCs on adolescent health care disparities is limited.

 Research on SBHCs indicates that users of such services are more likely than nonusers to be “high-risk” students and that frequent users are most often seeking care for chronic disease management or mental health services [99, 100]. Another study provided strong evidence that SBHCs can help reduce teenage childbearing in African American youths [101]. A more recent study of SBHCs serving predominantly African American students found that those who used the services were no more likely than nonusers to lack health insurance, and between 10 and 20% of the parents of the users reported they had difficulty obtaining community care for their child [102]. The findings also showed that students at the schools with SBHCs were significantly more likely than students at comparable schools without SBHCs to have visited a medical provider during the study period and were two-and-one-half times more likely to have seen a mental health provider. Additionally, the findings indicated that their SBHCs asthmatic students were half as likely to have an emergency department visit as the children in schools without SBHCs. The authors noted that SBHC care seemed to largely substitute for care which is normally provided by community-based medical and dental providers [102]. 

 School health services are widely recognized as important to child well-being. School health services were originally designed to supplement and not supplant community health care providers of health services for youths. The School Health Policies and Program Study (SHPPS) of 2006 found that nationwide 81.5% of schools had a coordinator of health services, 86.3% of schools had either a full-time (35.7%) or a part-time nurse (50.6%), and only 15.7% of schools had a school physician [103]. Yet, these services, because of easy access and lack of financial charges, could help provide access to care for racial and ethnic urban students and help reduce health care disparities. However, a recent analysis of the SHPPS data found that the higher the proportion of racial/ethnic minority students the less likely health services were offered to students in those schools [104]. 

## 11. Dental Insurance and Oral Health

Dental caries is the most common chronic disease among children in the United States [105]. Untreated dental caries can lead to chronic pain, inability to eat, compromised nutritional status, missed school days, and diminished self-esteem [106, 107]. The primary reason for inadequate access to dental care for children is lack of dental insurance. Without adequate access to preventive and restorative dental services children are at substantial risk for oral health disease, including loss of teeth. The greatest oral disease burden occurs in lower socioeconomic status and racial/ethnic minority groups [108]. A poignant example of this occurred in February 2007 when an economically disadvantaged African American boy who is 12 years old died from a severely infected dental carries. *The Washington Post* wrote that a routine $80 extraction would have likely saved his life [109]. 

 Two studies, using the National Survey of Children's Health, a nationally representative data set, found that 77% of children had dental insurance; of these children, 29% had public dental insurance. Public dental insurance is provided as a mandated benefit for Medicaid-eligible children through the Early and Periodic Screening, Diagnosis, and Treatment (EPSDT) program and states have the option to include dental care as part of the Child Health Insurance Program (CHIP), which all states but Delaware have agreed to do [110]. About 23% of all children were uninsured for dental care in 2007. In other words, children were 2.6 times more likely not to have dental insurance as they were not to have health insurance. White children had the highest proportion (63%) of children with private dental insurance, while African American children had the highest proportion (43%) with public dental insurance [110, 111].

 In relation to race/ethnicity, Hispanic children were least likely to ever have seen a dentist or had a dental visit in the previous year. However, while African American children were most likely to have dental insurance, they had the greatest disparity relative to insurance status to not receiving preventive dental care in the previous year. African Americans of all socioeconomic strata are most likely than any racial/ethnic group to reside in segregated communities [112]. The availability of professional dental care in neighborhoods where African American children reside may be limited and may serve as an impediment to some for access to dental care. The proportion of practicing dentists in the United States that are African American (5%) or Hispanic (<1%) is very limited [113]. In addition, white (58%), African American (46%), and Hispanic (64%) parents who reported that their children had not received all the dental care needed reported that it was because it “costs too much” and “no insurance” or “health plan problems”. [113]. Thus, it should not be surprising that both African American and Hispanic children with public dental insurance were more likely to have had preventive dental care than if they had private dental insurance. Private insurance programs have more exclusions, deductibles, and copays than public insurance programs. Furthermore, due to more generous dental reimbursement rates, children with CHIP dental coverage appear to have better access to dental care than children covered by Medicaid. However, regardless of race/ethnicity not having dental health insurance reduces by half the probability a child to have received preventive dental care, and this effect is more dramatic for African American children [114].

## 12. Conclusions

 The purpose of this review was to highlight the multiple health disparities afflicting American youth. Acknowledging the variety of individual health issues and the disparities they create between racial/ethnic minorities and white youths is essential but not sufficient to change. There are tangible steps that must be taken to overcome the barriers keeping certain racial/ethnic groups with higher levels of comorbidities. In virtually all areas explored within this review there were significant differences among racial/ethnic groups. In most areas racial/ethnic minority groups were at an increased risk of being diagnosed with a particular chronic ailment. However, differences by socioeconomic status impacts health disparities as well. Most chronic disease differences were not simply based on racial/ethnic minority status but instead on socioeconomic status.

Access to care (i.e., health insurance) is also a main concern in health disparity research. The majority of children that are uninsured are racial/ethnic minorities. There are many issues with enrollment that keep the numbers of children participating much lower than they should be. This issue spills over into prescription drug use, in which racial/ethnic minorities and uninsured children fill a fraction of the prescriptions given to nonminority, insured children. To prevent groups from becoming more disparate in health status, steps need to be taken to reduce the number of uninsured children. There are several options, including universal child healthcare, easing the enrollment process for public programs such as CHIP or Medicaid, or increasing healthcare availability within the public school system. Children that utilize school-based healthcare are often more at risk, so it is important that they find service somewhere. By increasing these points of service and the quality of care provided, a large number of underserved racial/ethnic minorities will be helped.

When discussing healthcare, many times dental health is overlooked; this should not be an optional form of health care. Oral health is incredibly important to an individual's current and future health status. Many health issues may be exacerbated by oral healthcare problems. By adding dental care coverage to insurance programs available to Medicaid and CHIP recipients, all disenfranchised youths will be healthier. This coverage should automatically be added to health insurance plans, both public and private to ensure oral health care for all youths.

All of the conditions examined in this review may be influenced by environmental and other external factors. We chose to review access to health services (including health insurance, dental insurance, and school-based health services) to help reduce the chronicity of diseases. When speaking about access to health care, one should not narrowly construe the concept as only having access to health insurance. It should also include appointment accessibility and the availability of evening and/or weekend hours. Another barrier to health care access is the proximity of the service or the provider to the patient. These barriers are often detrimental in the process of seeking appropriate health care among racial/ethnic minorities. Additionally, many socioeconomically disadvantaged groups have low health literacy. This means that they are unaware of the opportunities available to them and how to access them. If they are unaware that those services exist and that they are eligible for them, it is unlikely that they will take advantage of them. Another reason for racial/ethnic disparities is the notion of institutional racism that exists and prevents minorities from gaining access to and using health care. Overall, for a nation of great wealth and prosperity, our children should be our number one concern, and following some of these suggestions will help alleviate the chronic disease disparities that currently exist.

Many chronic diseases share common risk factors that if adequately addressed in broad-based prevention programs could greatly reduce the onset of many chronic health conditions. Among the targeted interventions would be the following: continued reduction in smoking; reducing consumption of poor diets (i.e., high fat and high sodium) at home and in schools; increasing the required physical education in schools to help learn life-long exercise skills; offering comprehensive health education programs K-12 grades to help create a health literate society and avoid harmful substances, including alcohol and other drugs; and increasing early access to comprehensive health care to help prevent exacerbation of illnesses which could develop into life-long impairments. Further elaboration of needed interventions to reduce racial and ethnic disparities can be found elsewhere [114, 115].

As a nation, we have made great strides in reducing mortality from chronic diseases. However, the rate of chronic disease development has not substantially changed. The result is that in 2003 the United States spent $227.0 billion on just 7 chronic diseases (i.e., cancer, hypertension, stroke, mental disorders, pulmonary conditions, heart disease, and diabetes). Additionally, businesses lost $1.1 trillion in missed workdays and lower productivity related to health care problems [116]. Our current national economic crisis is, in part, tied to our ability to maintain a healthy workforce. “While it is well understood among policy makers that economic growth is dependent on investments in human capital, the importance of good health in maintaining a competitive work force is frequently ignored” [116]. Targeting the health of youths is one of the most effective cost-benefit means of reducing chronic diseases in society.
